# Ultrasound elastography in pediatric care: bridging innovation and noninvasive diagnostics

**DOI:** 10.1186/s13052-026-02279-6

**Published:** 2026-06-02

**Authors:** Martina Piersanti, Federica Ferrari, Marisa Piccirillo, Rino Agostiniani, Marta Zerunian, Damiano Caruso, Fortunata Civitelli, Marco Graziani, Pasquale Parisi, Elisabetta Cortis, Giovanni Di Nardo, Maurizio Mennini

**Affiliations:** 1 Pediatrics Unit, Neuroscience, Mental Health and Sense Organs (NESMOS) Department, Faculty of Medicine and Psychology, Sapienza University of Rome, Via Di Grottarossa 1035-1039, 00189 Rome, Italy; 2 UOC of Pediatrics, Sant’Eugenio Hospital, Rome, Italy; 3 The Italian Pediatric Society, 00100 Rome, Italy; 4 Department of Pediatrics, San Jacopo Hospital, 51100 Pistoia, Italy; 5 Radiology Unit, Department of Medical-Surgical Sciences and Translational Medicine, Sant’Andrea University Hospital, 00189 Rome, Italy; 6 Pediatric Gastroenterology and Endoscopy Unit, Department of Pediatric Specialties, Santobono Pausilipon Children’s Hospital, Naples, Italy

**Keywords:** Ultrasound elastography, Pediatric diagnostics, Shear wave elastography (SWE), Liver fibrosis, Noninvasive imaging

## Abstract

This review explores the application and limitations of ultrasound elastography (USE) in the pediatric population, addressing its diagnostic value across different organs and suggesting future directions for research and clinical practice. A literature review was conducted using PubMed to identify studies published between 2010 and 2025 that examined USE applications in children. The search focused on studies assessing the liver, kidney, bowel, pancreas, muscle, connective tissue, and lung. The review highlights the established clinical application of USE in the assessment of liver fibrosis. It also provides an overview of new potential applications in kidney diseases, inflammatory bowel disease, cystic fibrosis, and muscle disorders. Two-dimensional shear wave elastography (2D-SWE) demonstrates excellent diagnostic accuracy in liver disease. However, in kidney applications, variability in results and the lack of standardized protocols limit clinical utility. SWE has shown promise in in the early detection of pancreatic insufficiency and muscle stiffness in cerebral palsy. Emerging applications include monitoring lung fibrosis and assessing fetal lung development. Despite its advantages—being noninvasive, repeatable, and well tolerated—USE remains limited by operator dependency, lack of standardization, and poor reproducibility in specific organs remain a major challenge. USE demonstrates significant potential for noninvasive diagnostics in pediatric care, especially in reducing invasive procedures and improving patient follow-up. However, further studies are needed to establish standardized protocols, reliable cut-off values, and broader applications across different organ systems. Integration of USE with other imaging modalities and utilizing advancements in artificial intelligence may enhance its diagnostic yield.

## Introduction

### Definition of principles of Elastography

USE is an imaging method based on tissue stiffness, relying on the propagation of high-frequency mechanical waves [1].

Tissue stiffness refers to its resistance to deformation under force, measured by stretching the material. “Deformation” indicates the change in length relative to the original length, while “stress” refers to force per unit area [2]. The strain ratio, a semi-quantitative stiffness index, compares the strain of a lesion within a region of interest (ROI) to that of an adjacent normal area. A ratio > 1 suggests the target lesion is less deformable and stiffer [2].

Tissue elasticity, defined as resistance to deformation and recovery after force removal, follows Hooke’s law: σ=Γ∙ε, where stress (σ) is the force per unit area, deformation (ε) is the strain, and the elastic modulus (Γ) relates stress to deformation [3].

Three elastic moduli are used to characterize tissue deformation:

1. Young’s modulus (E): normal stress produces a regular strain, with values varying by tissue (e.g., liver: 0.4–6 kPa, bone: 10 kPa) [2, 4].
2. Shear modulus (G): shear stress produces a tangential strain [4].
3. Bulk modulus (K): internal pressure causes volume deformation [4].

Elastography primarily estimates tissue stiffness using Young’s modulus, with higher values indicating greater stiffness [4].

USE techniques differ based on methods of tissue excitation [4]:

1. Strain imaging methods: also called static methods, they use external or internal compression to measure normal stress and strain.
2. Shear wave imaging (SWI): also called dynamic methods, they employ ultrasound-generated shear waves via mechanical vibrations or acoustic radiation force impulse (ARFI) [4].

### Technological evolution of elastography and clinical applications

Elastography, first described in the 1990s, evaluates tissue stiffness and has demonstrated over the years its clinical relevance for diagnosing and monitoring chronic diseases across anatomical regions [1]. USE has become increasingly applied in both clinical and research settings for the evaluation of multiple organs in children, including the brain, muscles, connective tissue, spleen, pancreas, bowel, lungs, kidneys, skin, lymphatic tissue, and especially the liver, its primary application [5]. This review focuses on the application of USE in the liver, kidneys, bowel, pancreas, muscles, and soft tissues, highlighting the most commonly used techniques. USE detects changes in tissue mechanics, making it valuable for assessing fibrosis, inflammation, and neovascularization key pathological processes in tissue alterations [6]. The methods differ based on the external mechanical stimulus: strain-based elastography uses probe pressure or endogenous forces, while SWE involves shear waves induced by the imaging system [6].

## USE techniques (table 1)

**Table 1:** Main elastography techniques. TE: transient elastography; pSWE: point shear wave elastography; 2D-SWE: two-dimensional shear wave elastography; ROI: region of interest; SWV: shear wave velocity; ARFI: acoustic radiation force

Technique	Physical Principle	Advantages	Limitations	Pediatric use
TE	Measures SWV generated by low-frequency mechanical vibration transmitted through tissue	- Rapid and easy to perform, − Good reproducibility in liver − Widely validated for fibrosis assessment	− No anatomical imaging guidance − Limited in obesity and ascites - Poor repeatability in children (motion-related variability) − Limited to liver evaluation	− Liver fibrosis assessment - Steatosis evaluation (CAP) - Portal hypertension (indirect use)
pSWE	Uses ARFI to induce localized shear waves and measure their propagation speed in a defined ROI	− Can be performed during standard US exam - ROI-based quantitative assessment − Applicable to deeper organs	- Operator-dependent ROI selection − Limited sampling area - Affected by anisotropy and vascularization (especially kidney)	− Liver fibrosis - Kidney stiffness (research setting) - Pancreas (CF, T1DM) − Muscle stiffness
2D-SWE	Generates multiple shear waves in real time to create a 2D quantitative stiffness map	- Real-time visualization − Larger sampling area - Higher diagnostic accuracy (especially liver) − Better spatial representation of stiffness	- Device-dependent variability − Requires standardization of protocols − Potential artifacts in deep or heterogeneous tissues	- Liver fibrosis (most validated) − Pancreas − Muscle and soft tissues - Emerging applications (lung, bowel)

**Fig. 1:**
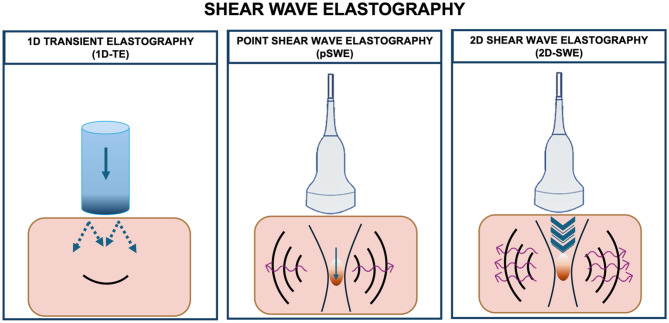
Graphic representation of different types of SWE described in the text including 1D-TE, pSWE and 2D-SWE

There are two main significant types of USE, which are SWE and strain (SE) elastography [7] (Fig. 1).

### SWE

SWI evaluates the propagation of shear waves, which travel perpendicular to tissue displacement, unlike compression waves propagating in the same direction [3]. Unlike strain imaging, which measures tissue displacement parallel to the applied normal stress, SWI relies on dynamic stress to generate shear waves traveling parallel or perpendicular to the stimulus [4]. The first commercially available SWI system, FibroScan™ (Echosens, Paris, France), was developed for liver assessment [3]. In SWI, wave speed correlates with tissue stiffness: faster in rigid tissues and slower in soft tissues. Acoustic waves, which are longitudinal compression waves, travel rapidly through tissues (1450–1550 m/s) and even faster in rigid materials but are challenging to track ultrasonically. Shear waves, in contrast, are transverse and propagate more slowly (1–10 m/s) [8].

Currently, SWI (which includes SWE) comprises three main technical approachesthree technical approaches:

1. Transient 1D elastography (1D-TE): the earliest SWI system for liver evaluation, which integrates an ultrasound transducer and vibrating mechanical device without B-mode image guidance [4].
2. Point shear wave elastography (pSWE): uses ARFI to generate shear waves by converting absorbed acoustic energy. Unlike 1D-TE, pSWE is performed on standard ultrasound machines using conventional probes [4].
3. 2D-SWE: the most advanced technique, capturing multiple focal areas sequentially to create a shear-wave cone, generating real-time quantitative elastograms and measuring velocity or Young’s modulus [4].

#### Transient Elastography (TE)

TE is a non-imaging elastographic technique based on pSWE and 2D-SWE combining imaging with elastography [7]. TE, specifically developed for liver assessment, generates low-frequency vibration pulses (50–500 Hz) transmitted via a piston with a single-element ultrasound transducer (FibroScan) without B-mode guidance [2]. FibroScan, also known as vibration-controlled transient elastography (VCTE), provides liver stiffness measurement (LSM) by measuring the velocity of elastic shear waves traveling through the liver (expressed in m/s) to estimate fibrosis severity [9].

FibroScan provides two key parameters: the controlled attenuation parameter (CAP), which quantifies liver fat (steatosis), and LSM, which assesses fibrosis [9].

TE is particularly useful for noninvasive liver stiffness assessment in pediatric patients, reducing the need for liver biopsies. However, Rowland et al. (2020) reported that TE shows poor accuracy in children due to random measurement variations, leading to low repeatability [10].

Different probes are available based on patients’ body mass index: M probes for most patients and XL probes for obese patients. Selecting the correct probe is essential, as it affects the accuracy of steatosis detection via CAP and liver stiffness measurement [11].

The FibroScan technique can be integrated with imaging modalities, such as ultrasound, for comprehensive liver assessment, enabling more accurate diagnosis and monitoring of liver diseases, particularly fibrosis and steatosis [12].

### Strain elastography (SE)

The first successful device for imaging tissue elasticity was SE, introduced in 1991 by Cespedes and Ophir [8]. In SE, an ultrasound transducer applies slight compression, and radiofrequency (RF) ultrasound signals are captured before and after compression. Deformation is more significant in soft tissues than stiffer ones, as tissue displacement is larger near the compression source and decreases with distance [8]. Strain imaging deformations using ultrasound encompass two approaches:

1. Strain elastography (SE): the operator manually compresses the tissue with the transducer. This method is effective for examining superficial organs, including the breast and thyroid, whilst challenging for deeper organs like the liver.
2. ARFI: the transducer remains stationary while tissue displacement is generated by internal physiological motion (e.g., cardiovascular or respiratory), properly assessing deeper organs [4].

Tissue displacement measurement along the applied stress direction can be achieved using RF echo correlation-based tracking, doppler processing, or a combination of both. The most practised method is RF echo correlation-based tracking, which evaluates tissue displacement by correlating RF echo signals between different search windows during multiple acquisitions [4]. The elastogram, a color-coded map overlaid on the B-mode image, visualizes deformation measurements: low deformation (stiff tissue) appears in blue, while high deformation (soft tissue) appears in red [4]. An alternative method is ARFI strain imaging, where a short, high-intensity acoustic pulse induces tissue displacement perpendicular to the surface. Displacement is visualized as an elastogram superimposed on the B-mode image [13].

### Limitations of USE

USE is widely employed in clinical settings for diagnosing and monitoring various diseases and has grown exponentially in recent years. Therefore, understanding its limitations is crucial for guiding future research and optimizing its clinical applications [4]. Like other operator-dependent techniques, such as free-hand ultrasound, USE faces limitations in obtaining objective measurements. The selection of the region of interest (ROI) is operator-dependent, leading to measurement variability. Additionally, the interpretation of artifacts, including shadowing, reverberation, and clutter, is subjective and can lead to confounding results. Tissue attenuation further limits the accuracy of measurements at greater depths, reducing the assessment capability for deeper organs [14].

Patient-specific factors also affect USE accuracy. In obese patients, subcutaneous fat attenuates the propagation of external stimuli, while in ascitic patients, subcutaneous fluid can invalidate measurements. Variability also arises from differences in commercially available systems, which use various external stimuli and non-uniform designs, complicating measurement comparisons across devices.

Strain elastography, which relies on external stimuli, suffers from poor reproducibility due to the difficulty in controlling applied stress during manual compression. Internal stress sources, such as cardiac and respiratory motion, further contribute to measurement variability, as physiological changes can influence results interpretation at different intervals [15]. Another significant limitation of USE is its inability to adequately capture tissue viscoelastic properties and structural heterogeneity; this affects the characterization of heterogeneous tissues, such as tumors, which may contain rigid fibrotic zones, calcifications, and softer regions like hemorrhages or cystic areas. The simplified elastic parameters of USE may not fully describe these complex regions [4].

Despite these limitations, USE remains a promising technology that is under active development. After summarising USE’s physical principles and core techniques, we will evaluate its performance in organs such as the liver, pancreas, kidney, intestine, lung, muscle, and soft tissue.

## Aim of the review

This review provides a comprehensive overview of the current applications of USE as a diagnostic modality and its potential utility in clinical practice and research in pediatrics.

A narrative literature review was conducted to identify and synthesize relevant evidence on the applications of ultrasound elastography in pediatric populations. A structured search was performed in PubMed for articles published between January 2010 and March 2025, using combinations of the following keywords: “ultrasound elastography”, “shear wave elastography”, “strain elastography”, “pediatric”, “children”, “liver”, “kidney”, “bowel”, “pancreas”, “lung”, and “muscle”.

The selection process was guided by relevance to the scope of the review, with particular emphasis on studies addressing clinical applications of USE in pediatric patients. Both original research articles and review papers were considered.

To ensure comprehensive coverage, the reference lists of selected articles were manually screened to identify additional relevant studies.

Studies were included if they reported data on the use of elastography in pediatric populations or provided clinically meaningful insights applicable to pediatric practice. Given the limited availability of pediatric-specific data in certain areas, selected studies conducted in adult populations were also included when their findings were considered potentially translatable to pediatric settings.

The selection of studies was not conducted according to a predefined systematic review protocol, and no formal quality assessment or risk-of-bias evaluation was performed, in line with the narrative nature of this review. Nevertheless, efforts were made to include studies with robust methodology and to critically appraise the available evidence, particularly in areas characterized by heterogeneity or conflicting results.

## Clinical applications in pediatrics (table 2)

**Table 2:** Applications of elastography in pediatrics. 2D-SWE: two-dimensional shear wave elastography; TE: transient elastography; pSWE: point shear wave elastography; CKD: chronic kidney disease; AGN: acute glomerulonephritis; SWE: shear wave elastography; ARFI: acoustic radiation force impulse; UC: ulcerative colitis; SE: strain elastography; CF: cystic fibrosis; CP: cerebral palsy; US: ultrasound; ILD: interstitial lung disease; [P]: pediatric; [A]: adult; [M]: mixed populations or extrapolated evidence

Organ/ System	Disease/Condition	Elastography Technique	Key Findings	Limitations
Liver [4, 16–22]	Fibrosis, steatosis, portal Hypertension	2D-SWE, TE, pSWE	-High diagnostic accuracy for fibrosis staging [P]; -Strong correlation with histological findings [P/M]	Limited by variability in cut-off values and reduced specificity in distinguishing intermediate fibrosis stages [P/M]
Kidney [23–28]	CKD, AGN, renal transplant	SWE, ARFI	-High potential for early CKD diagnosis [P]; -Strong correlation with renal dysfunction [P];	Limited by high inter-study variability [P/M], lack of standardized protocols [P/M], and influence of anisotropy and vascularization [P];
Bowel [29–34]	CD, UC	SWE, SE	-Moderate-to-good accuracy in detecting bowel fibrosis in inflammatory bowel disease [P/M]	Limited by small sample sizes, lack of histological validation [P], and inconsistent ability to differentiate fibrosis from inflammation [P/M]
Pancreas [35–41]	CF, type 1 diabetes	pSWE, 2D-SWE	-Early desease detection of pancreatic insufficiency and tissue softening in CF [P]	Limited by small study and technical challenges related to deep anatomical location [P]
Muscle [42, 43, 43–47]	CP	SWE	-Increased muscle stiffness detected in spastic conditions such as CP [P]	Limited by lack of standardized reference values and variability in acquisition protocols [P/M]
Lung [48–53]	ILD	SWE, Lung US	-Feasible assessment of lung stiffness with potential role in ILD evaluation [A/M]	Limited by small number of studies, lack of standardization, and reduced correlation with structural imaging findings [A/M]

### Liver (Figure 2)

**Fig. 2:**
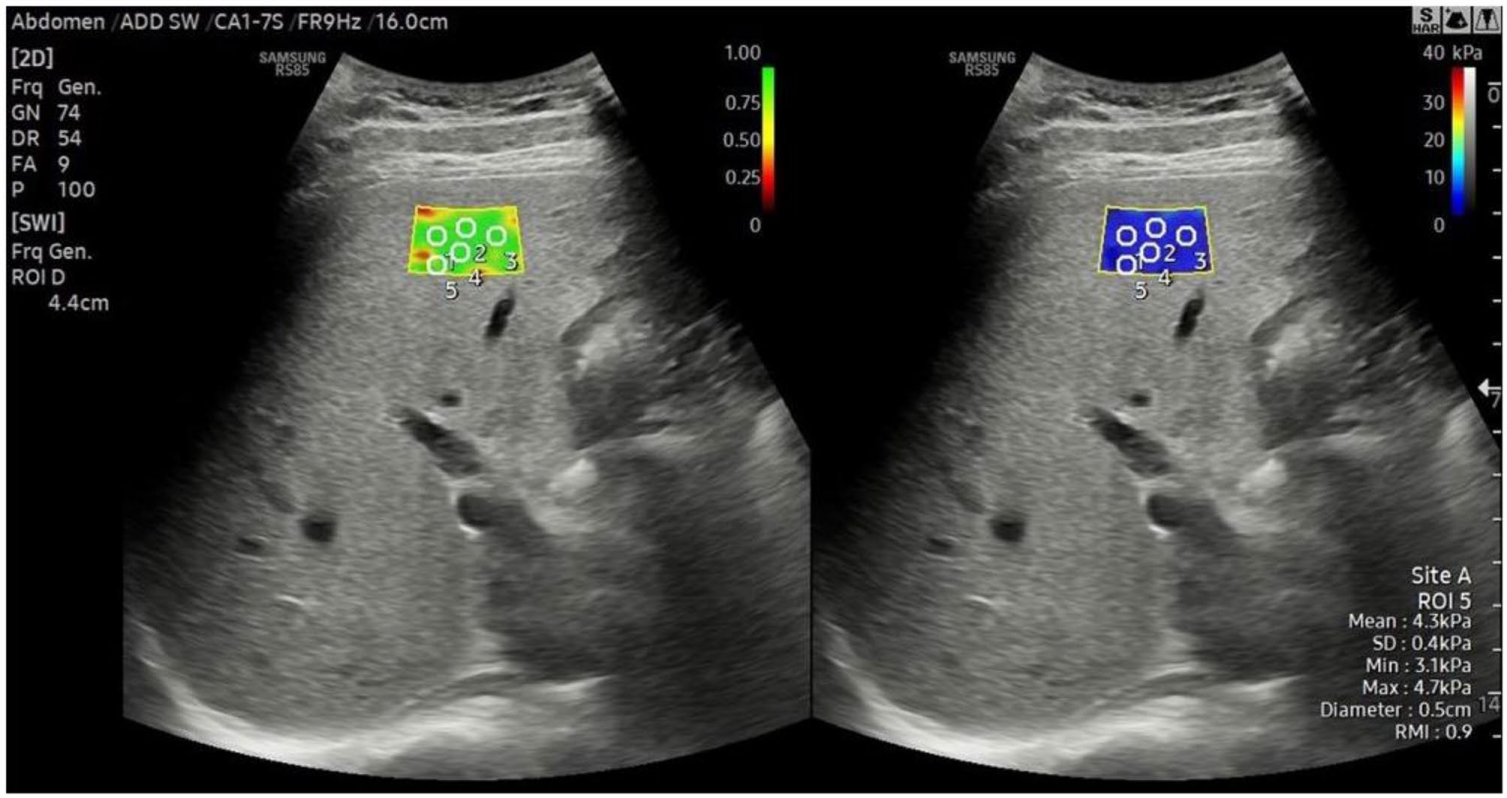
Example of a 15 year-old female underwent 2D shear wave ultrasound of the liver (Samsung prestige RS85 - Samsung Medison, Seoul, South Korea) with multiple measurements expressed as kPa

USE has emerged as an accurate non-invasive imaging method for liver assessment, becoming increasingly important for diagnosing cirrhosis, differentiating healthy from fibrotic tissue, and estimating liver stiffness in children. Various elastography techniques, including transient elastography (TE), point shear wave elastography (pSWE), and two-dimensional shear wave elastography (2D-SWE), can be used for the assessment of liver fibrosis [54]. In particular, 2D-SWE in liver has diagnostic accuracy superior to 1D-TE in the diagnosis of severe fibrosis and superior to pSWE in the diagnosis of significant fibrosis [4]

Magnetic resonance elastography (MRE) also provides liver stiffness measurements but has limitations due to its higher costs, reduced availability and the possible limitation related to claustrophobia [55].

**Pediatric liver diseases studied with USE:**

- Autoimmune hepatitis (AIH): TE demonstrates higher diagnostic accuracy for fibrosis compared with noninvasive serum-based indices, including the AST to Platelet Ratio Index (APRI), Fibrosis-4 Index (FIB-4), and the AST/ALT ratio [16]. This diagnostic role was further supported by Wu et al. (2019) [16]. However, a more recent study by Dillman et al. (2024) showed that magnetic resonance elastography (MRE) may provide additional accuracy, with strong correlations with histologic scores and laboratory markers [17].
- Cystic fibrosis (CF): As reported by Levitte et al. [56], MRE remains the preferred method compared to SWE, due to liver’s heterogeneity, responsible for lower reliability [56]. SWE correlates with fibrosis, but variable performance arises in patients with pathological MR findings, highlighting that sampling windows are more limited in USE. SWE is more effective when paired with the 6-MHz probe [56].
- Other autoimmune liver diseases (AILDs): TE outperforms serum biomarkers in primary biliary cholangitis (PBC) and primary sclerosing cholangitis (PSC) for fibrosis staging [57].
- Congenital disorders: TE has shown promising results in congenital liver disorders, including biliary atresia (BA), alpha-1 antitrypsin deficiency (A1ATD), and Alagille syndrome (ALGS). In the FORCE study [18], TE demonstrated a progressive increase in liver stiffness with advancing portal hypertension. However, in ALGS, differences in stiffness were less pronounced, likely reflecting its distinct pathophysiology. Across these conditions, liver stiffness measurements (LSM) showed strong correlations with laboratory markers such as total bilirubin, platelet count, and albumin [18].
- Portal hypertension (PH): PH, a significant complication of chronic liver disease (CLD), is challenging to assess noninvasively in children due to the limitations of techniques in estimating hepatic venous pressure gradient (HVPG). SWE has demonstrated higher success rates than TE in detecting clinically significant PH, as shown by Elkrief et al. [58]. Splenic stiffness measurement (SSM) via pSWE is a better predictor of varices than LSM, particularly in patients with extrahepatic portal vein obstruction [59].
- Nonalcoholic-associated fatty liver disease/Metabolic dysfunction-associated fatty liver disease (NAFLD/MAFLD): Pediatric fatty liver disease, often linked to obesity, can progress to steatohepatitis (NASH) and fibrosis [60]. The controlled attenuation parameter (CAP), derived from transient elastography, measures liver fat independently of fibrosis and has shown strong diagnostic performance: in a study conducted by Ferraioli et al. in 2017 in which US and CAP data were analyzed on 289 children, dichotomized CAP showed a performance of 0.70 (sensitivity, 0.72 and specificity 0.98), which was better than that of US (performance 0.37), and they concluded that a CAP cut-off value of 249 dB/m rules out hepatic steatosis with a very high specificity. [19]. Magnetic Resonance Imaging-Proton Density Fat Fraction (MRI-PDFF) offers higher sensitivity and specificity compared to CAP, with the advantage of being operator-independent [20].
- Post-transplant liver monitoring: USE is also being evaluated for real-time, noninvasive monitoring of liver stiffness post-transplant, reducing the need for biopsies. However, challenges such as device variability and the lack of standardized cut-off values limit its routine use, requiring further research [61].

#### Advantages and limitations of USE in liver assessment

2D-SWE is the most validated ultrasound technique for liver fibrosis., with diagnostic accuracy superior to 1D-TE and pSWE [4]. World Federation for Ultrasound in Medicine and Biology (WFUMB) guidelines recommend USE to distinguish significant (F ≥ 2) and advanced (F ≥ 3) fibrosis from non-significant fibrosis (F0–F1) [21]. However, it appears to have less specificity in differentiating individual stages of fibrosis, as highlighted by Medyńska-Przęczek et al. [22]. Overall, findings across studies are largely consistent in supporting the diagnostic value of elastography for liver fibrosis in pediatric populations. However, variability in reported cut-off values, differences in imaging techniques, and heterogeneity in patient populations limit direct comparability and hinder clinical standardization.

### Kidney

Compared to the liver, kidney stiffness is more challenging to assess using elastography, due to the kidney’s deep anatomical location and tissue heterogeneity. Shear wave velocity (SWV) measurements are highly sensitive to parameters like anisotropy and vascularization, requiring ultrasound-guided techniques for accurate renal assessment [23]

Studies have reported contradictory results regarding kidney stiffness and fibrosis, particularly in pediatric populations. This inconsistency across studies reflects differences in study design, measurement protocols, and patient characteristics, and highlights the current uncertainty regarding the clinical applicability of renal elastography. Desvignes et al. (2021) found excessive variability in SWE measurements among 95 children, including 31 kidney transplant recipients, concluding that no reliable correlation between elasticity and fibrosis could be established [24].

**Applications in Pediatric Kidney Diseases:**

- Acute glomerulonephritis (AGN): ARFI-e elastography demonstrated higher stiffness in children with AGN compared to healthy controls, highlighting its potential for early noninvasive detection [62].
- Obesity and renal stiffness: studies by Karaman et al. (2021) and Mocnik et al. (2023) confirmed that overweight and obese children exhibit increased renal cortical stiffness, suggesting that obesity worsens kidney stiffness, especially in chronic kidney disease (CKD) and hypertension [25, 26].
- Chronic kidney disease: Liu et al. (2021) measured Young’s modulus (YM) in children with CKD and found higher values correlated with renal dysfunction, demonstrating that SWE could serve as an early diagnostic tool [27].

#### Challenges in renal elastography

Kidney stiffness is influenced by multiple factors beyond fibrosis, including pressure, vascularization, and anisotropy. A 2022 review analyzing eight studies on pediatric CKD found inconsistent results: five studies reported higher SWV in patients, two showed lower SWV, and one found no significant difference. As previously discussed, variability in acquisition protocols and measurement conditions—covering kidney selection, pole location, posture, and ROI size—contributes to inconsistent findings in renal elastography [28]. Overall, the heterogeneity of available data and the absence of standardized acquisition protocols currently limit the reproducibility and clinical translation of renal elastography findings. Furthermore, some conclusions are extrapolated from adult studies, which may not fully capture the specific anatomical and physiological characteristics of the pediatric kidney.

### Bowel

The diagnosis of pediatric inflammatory bowel disease (PIBD), including ulcerative colitis (UC) and Crohn’s disease (CD), requires evidence of chronic gastrointestinal inflammation, typically confirmed through invasive endoscopy and biopsy [63]. Bowel ultrasonography is an established tool in the initial diagnostic work-up and in the follow-up of IBD for monitoring disease activity and early detecting complications such as stenosis, fistulae and abscesses. In the last decade several studies have evaluated the potential applications of USE in IBD, with a focus on stricturing CD.

#### USE in stenosis assessment in CD

Stenosis in CD are often the result of a combination of fibrosis and inflammation. In CD patients distinguishing between a primarily inflammatory or a fibrotic disease has a relevant impact on clinical decision making and on therapeutic strategies: patients with strictures characterized by prominent inflammation may be managed with medical treatment, whereas patients with a prevalent fibrotic stenosis, often require endoscopic dilatation or surgery. Accurate identification of stenosis typically involves endoscopy and cross-sectional imaging such as ultrasound, magnetic resonance imaging (MRI) or computed tomography scan (CT-scan) [64]. The noninvasive assessment of fibrosis using USE is appealing for clinical practice, in order to optimise the treatments for strictuing disease [29–32, 65].

USE imaging modalities:

- Strain elastography (SE): Fufezan et al. (2015) classified intestinal stiffness into three patterns using SE and hydrosonography (HS) in 14 pediatric CD patients: normal/remission (type A), inflammation (type B), and fibrosis (type C). The study found statistically significant correlations between SE strain ratios, clinical markers of disease activity, and imaging techniques (hydrosonography and magnetic resonance enterography) [30]. However, it lacked histopathological validation.
- SWE: Chen et al. (2024) showed SWE to be an independent predictor of disease progression in 130 adult CD patients with non-stenotic disease (B1 phenotype). During a 33-month follow-up, 20% of patients progressed to structuring (B2) or penetrating (B3) disease [31].
- Contrast-enhanced ultrasound (CEUS) with SWE: Sidhu et al. (2023) investigated 25 pediatric CD patients with terminal ileum involvement. CEUS effectively identified bowel fibrosis in patients requiring surgery, but SWE showed no correlation with fibrosis severity [29].

#### USE in UC

Goertz et al. explored ARFI elastography for assessing colon and terminal ileum stiffness in patients with UC, but high variability and standard deviation were reported, suggesting a need for further studies [32].

Challenges in IBD:

USE may represent an innovative, non-invasive, promptly available, ancillary technique in the evaluation of intestinal fibrosis in IBD. From the preliminary available data, an overall moderate-to-good accuracy of USE in detecting fibrosis at histology was found. As concerns USE accuracy, point-SWE was found to perform better compared with SE and ARFI by Ding et al. As highlighted in the general limitations, the available evidence is limited by small sample sizes and heterogeneity in methodologies. Additionally, several findings are derived from adult populations, which may not be directly applicable to pediatric patients. [33, 34]

### Pancreas

USE offers potential applications for assessing pancreatic tissue stiffness, particularly in chronic conditions like CF. USE could be used to determine the strain index (SI) at various pancreatic sites, but manual compression is often ineffective due to the retroperitoneal location of the pancreas and small size. Aortic pulsations are commonly utilized as an alternative compression method, achieving optimal results when the pancreas is located between the probe and the aorta [35].

A study by Ozturk et al. (2017) established normal pancreatic elasticity values in healthy children, demonstrating that these values vary with age, weight, height, and BMI. This provides a baseline for assessing pancreatic stiffness in disease states [35].

#### Applications in CF

CF is an autosomal recessive disease associated with pancreatic insufficiency and the formation of pancreatic cysts due to altered secretions [66]. Non-invasive USE techniques, particularly pSWE and 2D-SWE, are preferred for pancreatic evaluation.

- Early Diagnosis of Pancreatic Insufficiency: in a 2022 study by Yilmaz et al., pSWE measurements were performed on 55 CF patients and 60 healthy children. Lower SWE values were found in CF patients compared to healthy controls, suggesting that pSWE may help detect pancreatic insufficiency early, even before clinical symptoms arise [36].
- Pancreatic Tissue Changes: CF-related pancreatic tissue softening is linked to adipose tissue replacement, resulting in reduced pSWE values. Studies, including one by Pfahler et al., confirmed significantly lower pancreatic pSWE values in CF patients compared to healthy controls [37, 38].

#### Applications in type 1 diabetes mellitus (T1DM)

Strain imaging has shown increased strain ratios in children with T1DM, suggesting its use as an early diagnostic tool. SWE measurements were found to correlate with the duration of T1DM, particularly in patients with complications like nephropathy and neuropathy [39, 40].

Another study conducted by Gupta et al. in 2024 showed that SWE values in children with DM1 correlated with body mass index (BMI), glycemic control, disease duration, and gamma-glutamyl transferase (GGT) levels. Children with DM1 had higher liver stiffness compared to controls, highlighting elastography’s potential role in monitoring metabolic impacts on liver health [41].

#### Limitations and future directions

Although promising, UE in pancreatic evaluation requires further research to fully validate its clinical utility. Although these studies suggest a potential role for elastography in pancreatic diseases, the evidence is still limited and largely based on small cohorts, reducing the generalizability of the findings. Further studies are needed to confirm these findings in larger pediatric populations. Its current applications focus on the early diagnosis and follow-up of conditions such as CF and T1DM, but future studies are needed to determine its broader effectiveness [5].

### Muscles and soft tissues

Diagnosing muscle diseases in children can be challenging, with standard methods often being invasive. USE provides a noninvasive alternative for assessing muscle stiffness and stretch. A study by Berko et al. showed that muscle stiffness increases with strain, stretch, and exercise, with greater changes observed in younger children [42].

#### Application in infantile cerebral palsy (CP)

CP is a group of permanent motor disorders caused by brain damage during early fetal brain development. A common consequence is muscle spasticity, traditionally measured using the Modified Ashworth Scale (MAS), which lacks quantitative accuracy, limiting its use in treatment planning, particularly for botulinum toxin injections [67, 68]. A study by Lallemant-Dudek et al. evaluated SWE in 16 CP patients and 29 healthy controls, measuring muscle stiffness in the gastrocnemius and biceps brachii at rest and under passive stretch. Results confirmed that spastic muscles were stiffer than healthy ones, with SWE providing a quantitative assessment of stiffness, and helping plan precise and localized treatments [43]. Several other studies have further emphasized that SWE detects increased SWV in spastic muscles, including alterations in muscle properties following botulinum toxin injections [43–45]. These findings suggests that SWE is a promising tool for assessing and tailoring treatments in CP patients.

#### Soft tissue lesions

UE also shows potential in differentiating between benign and malignant soft tissue masses based on stiffness. However, SWE alone lacks sufficient diagnostic accuracy. Sandomenico et al. demonstrated that combining elastography with Doppler ultrasound significantly increases diagnostic specificity, reducing false positive results [46]. Current evidence emphasizes the need for an integrated imaging approach, as histologic analysis remains the gold standard for diagnosing soft tissue tumors [46, 47].

#### Limitations and future directions

Although promising, USE has not yet been established in routine clinical practice for muscle and soft tissue evaluation. Its current applications focus on monitoring spasticity in CP and differentiating soft tissue lesions, but despite encouraging results, clinical application is still limited, reflecting the broader challenges of standardization and reproducibility in elastography. [5]. USE holds potential for noninvasive assessment in pediatric muscle and connective tissue diseases. Its role in the management of CP and muscle stiffness is particularly promising, but more research is necessary to confirm its diagnostic and therapeutic utility.

### Lung

Interstitial lung disease (ILD) is a common complication of connective tissue diseases (CTD) and is associated with increased lung stiffness and reduced survival. The current diagnostic gold standard is high-resolution computed tomography (HRCT), which provides imaging but does not measure lung elastic properties. USE, particularly SWE, may offer a noninvasive and reproducible alternative for assessing lung stiffness and ILD severity [48].

#### Feasibility of SWE in the lungs

Zhang et al. (2016) demonstrated that SWE is feasible for measuring lung elasticity, using surface waves to assess superficial lung tissue while avoiding ultrasound radiation force due to potential tissue injury from lung air content [49]. Lung US is widely used for detecting lung abnormalities such as thickening, pneumothorax, and cystic formations. SWE builds on this by combining imaging with lung elasticity measurements, potentially enhancing diagnostic utility [50]. A 2022 study by Huang et al. demonstrated high intra- and interobserver reliability of SWE measurements at the middle anterior lung site, distinguishing patients with ILD from healthy controls [48]. However, SWE’s diagnostic efficacy was lower in studies focused on pleural line evaluation, as measurements of Young’s modulus and SWV showed weak correlations with pleural line thickness [49, 50]. In the adult population, combining lung US with HRCT has shown promise. A prospective study reported 92% sensitivity and 89% specificity for lung US SWE in differentiating healthy individuals (F0) from those with ILD (F1–F3) [51]. However, results across studies are not entirely consistent, particularly regarding the correlation between elastographic parameters and structural lung changes, reflecting technical and methodological limitations. It should be noted that a substantial portion of the available evidence originates from adult studies, and its applicability to pediatric populations remains to be fully established.

#### Emerging applications: fetal lung development and pneumonia

- Fetal lung development: elastography has been used to monitor lung maturation in fetuses. Elasticity values increase during early gestation and decrease in later stages, correlating with the transition from dense interstitial cells to a single-layer respiratory epithelium in the alveoli [52].
- Pulmonary infections: lung elastography has shown potential in assessing changes in tissue stiffness caused by pneumonia and other lung conditions. However, standardization of image acquisition and reference values for lung elasticity are still needed before clinical adoption [53].

#### Limitations and future directions

Despite its potential, the use of USE in the lungs remains limited. The absence of standardized protocols and the limited number of studies restrict current clinical applicability. Further research is essential to establish normal lung elasticity values and refine its clinical applications for ILD and other pulmonary conditions. Moreover, much of the available evidence is derived from adult populations, and its applicability to pediatric patients remains to be fully established.

## Advantages and limitations of USE

The clinical applications of elastography differ significantly between adults and children due to anatomical and physiological variations. Most studies have focused on adults, as the technique was initially developed for this population [1]. The limited pediatric studies are primarily due to the challenges posed by children’s greater mobility, lower compliance during imaging, and difficulty in maintaining the required position [4]. Additionally, elastography’s early applications targeted liver fibrosis, a condition more prevalent in adults, while its use in children is often limited to specific cases like metabolic or congenital liver diseases [9].

### Anatomical and physiological differences

- Fat and connective tissue distribution: children have a higher and more uniform distribution of subcutaneous fat compared to adults, where visceral fat accumulation is more common and hormonally influenced [69]. This variation affects the propagation of pressure waves during elastographic assessments.
- Extracellular matrix composition: children have greater extracellular matrix content, with higher collagen, elastin, and vascularization to support growth [70]. As a result, connective tissue in children is more elastic and less fibrotic, complicating the interpretation of stiffness measurements. For instance, liver stiffness is naturally lower in infants and young children, potentially delaying early diagnosis of fibrotic diseases.
- Vascularization and organ location: higher vascularity in pediatric tissues, such as the pancreas, can affect elastographic readings, particularly given the pancreas’ retroperitoneal location and the influence of surrounding structures [35].

Understanding these differences is critical for accurate elastographic evaluation and for adapting diagnostic criteria to pediatric-specific conditions.

### Benefits compared to invasive methods

One of the main advantages of USE is its noninvasiveness, as all other ultrasound methods. The primary use of USE is to assess tissue stiffness, a parameter otherwise assessable by tissue biopsy. However, biopsy is a painful technique, requiring sedation or anesthesia, which may cause anxiety or emotional discomfort in children. It also requires longer preparation and finalization time and may lead to complications, such as scarring, infection, or bleeding. A technique that is well tolerated by children is also more accepted by families than invasive methods, as they are generally agreed upon with parents with minor concerns and apprehension [5]. The application of USE can obviate these issues [71].

In addition, being ultrasound-based, elastography is considered a safe technique, since it does not expose children to ionizing radiation, and provides real-time imaging [5].

An additional advantage of noninvasiveness is repeatability: examinations can be repeated frequently to follow the evolution of a disease or assess response to treatments, without causing damage to the patient [5, 71].

Consequently, the technique can be considered convenient for costs due to its noninvasiveness: by avoiding anesthesia, hospitalization, and management of complications, the overall costs are lower, along with the fact that the equipment is generally less expensive than that of other imaging technologies [5, 6]

### Limitations in pediatric population

While USE is widely used in clinical settings, it has several limitations, especially due to its operator-dependent nature. The following limitations summarize the main challenges of USE across different organ systems, many of which have been highlighted in the previous sections. Key challenges include subjective measurements, artifact interpretation, signal attenuation in deep tissues, and limited reproducibility due to different commercial systems and external stimuli [4, 14, 15]. Additionally, the method does not account for tissue viscoelasticity, which can affect diagnostic accuracy.

Challenges in Pediatrics:

1. Lack of standardization: there are no standardized protocols in pediatric USE, making it difficult to compare results across operators or centers. The limited clinical experience in pediatrics further impacts accuracy, as correct transducer positioning and data interpretation are operator-dependent [5].
2. Patient factors: infants and young children often have difficulty maintaining stable positioning during imaging, affecting image quality. Breathing, movement, bowel activity, meteorism, and excess subcutaneous fat contribute to artifacts, reducing reproducibility, particularly in obese children [72].
3. Safety considerations: the high-intensity ultrasonic pulse used in elastography may increase the thermal and mechanical indices, posing potential risks, especially in children with more vulnerable tissues [5].

Organ-Specific Challenges:

Limitations in the application of USE for specific organs, such as the liver, kidney, and lung, are reviewed in their respective sections. For example, while obesity, which has a high incidence in pediatric patients, complicates liver elastography, it also highlights its potential benefit for this population [72].

## Future outlook

USE has recently gained increasing traction in pediatric clinical settings, initially applied to liver studies and now expanding to other areas. Recent advancements include integrating USE with complementary imaging techniques, such as CEUS, magnetic resonance imaging (MRI), and computed tomography (CT). These combinations provide enhanced anatomical detail, improving diagnostic accuracy for interventions like biopsies and tumor ablations.

Advancements in Liver Elastography:

- Three-dimensional magnetic resonance elastography (3D MRE): compared to USE, MRE samples larger areas of the liver, reducing measurement variability and offering high diagnostic accuracy for fibrosis staging [73]. However, limitations include sensitivity to respiratory motion, high costs, reduced availability, and possible claustrophobia. Studies suggest 3D MRE, combined with proton density fat fraction (PDFF), improves diagnostic accuracy in liver disease staging [73].
- Artificial intelligence (AI): AI is being explored to reduce measurement variability and improve workflow efficiency in elastography applications [74]. Artificial intelligence may play a key role in improving standardization and reproducibility of elastography measurements. Machine learning algorithms could support automated image interpretation, reduce operator dependency, and enable more consistent quantification across centers. In the future, AI-driven tools may facilitate clinical decision-making by integrating elastography data with other imaging and clinical parameters.

### Emerging fields in pediatric elastography

Although primarily used for liver, pancreatic, and muscle evaluations, other emerging applications are under investigation in pediatrics:

- Urinary tissue stiffness: studies are investigating its role in conditions like nocturnal enuresis [75, 76].
- Thyroid gland evaluation: SWE is being investigated for differentiating benign from malignant thyroid nodules [77, 78].
- Brain tissue: elastography may aid in tumor characterization, particularly for gliomas [79].

## Conclusions

USE represents a groundbreaking and rapidly evolving technique with significant potential in both adult and pediatric clinical practice. Its inclusion in pediatric care could address several challenges, enhancing diagnostic accuracy, facilitating regular follow-up through its repeatability and noninvasiveness, and reducing reliance on invasive procedures. This, in turn, can lead to better patient and parent compliance, while simultaneously lowering hospital costs. While it is widely used in adults, especially for liver diseases, its routine use in pediatrics is still limited and requires further validation. This is largely due to the need for studies evaluating its feasibility, reproducibility, and reliability across different organs and clinical contexts. To overcome these limitations and enhance its application in pediatrics, following key steps must be taken:

- Standardization of measurements: establishing reproducible cut-off reference values will be crucial for ensuring consistent, reliable diagnostics in children. Standardized protocols across centers will minimize inter-operator variability.
- Training and expertise: as elastography is highly operator-dependent, thus investing in clinician training is essential to ensure accurate measurements and reliable data interpretation.
- Work organization: standardized workflow across centers would be useful to create to study more patients with USE and at the same time to select cases that need further investigation (e.g. MRE).
- Expanding clinical applications: future studies should explore elastography’s potential in other body systems, such as the lungs, pancreas, muscles, and soft tissues, to broaden its role in diagnosing and monitoring pediatric diseases.

With its potential to serve as a cost-effective, noninvasive diagnostic tool, USE could profoundly enhance pediatric care in the future. This review aimed to offer a comprehensive overview of its current applications, highlight its limitations, and propose pathways for future research, ultimately paving the way for its expanded integration into clinical practice.

## Data Availability

Not applicable.

## Abbreviations

USE: Ultrasound elastography
ROI: Region of interest
2D-SWE: Two-dimensional shear wave elastography
SWE: Shear wave elastography
SWI: Shear wave imaging
TE: Transient elastography
SE: Strain elastography
1D-TE: Transient 1D elastography
pSWE: Point shear wave elastography
ARFI: Acoustic radiation force impulse
VCTE: Vibration-controlled transient elastography
LSM: Liver stiffness measurement
CAP: Controlled attenuation parameter
BMI: Body mass index
MRE: Magnetic resonance elastography
MR: Magnetic resonance
RF: Radiofrequency
AIH: Autoimmune hepatitis
APRI: AST to platelet ratio rndex
FIB-4: Fibrosis-4 Index
AST/ALT: Ratio of the serum aspartate to alanine amino-transferase levels
AILDs: Other autoimmune liver diseases
PBC: Primary biliary cholangitis
PSC: Primary sclerosing cholangitis
BA: Biliary atresia
A1ATD: Alpha-1 antitrypsin deficiency
ALGS: Alagille syndrome
CF: Cystic fibrosis
PH: Portal hypertension
CLD: Chronic liver disease
HVPG: Hepatic venous pressure gradient
SSM: Splenic stiffness measurement
NAFLD: Nonalcoholic-associated fatty liver disease
MAFLD: Metabolic dysfunction-associated fatty liver disease
NASH: Steatohepatitis
MRI-PDFF: Magnetic resonance imaging-proton density fat fraction
dB/m: Decibels per meter
WFUMB: World Federation for Ultrasound in Medicine and Biology
SWV: Shear wave velocity
CKD: Chronic kidney disease
AGN: Acute glomerulonephritis
YM: Young’s modulus
SI: Strain index
PIBD: Pediatric inflammatory bowel disease
UC: Ulcerative colitis
CD: Crohn’s disease
CT-scan: Computed tomography scan
CEUS: Contrast-enhanced ultrasound
CF: Cystic fibrosis
US: Ultrasound
GGT: Gamma-glutamyl transferase
T1DM: Diabetes mellitus
CP: Cerebral palsy
MAS: Modified Ashworth scale
ILD: Interstitial lung disease
CTD: Connective tissue diseases
HRCT: High-resolution computed tomography
3D MRE: Three-dimensional magnetic resonance elastography
AI: Artificial intelligence
